# Qualitative and quantitative evaluation of hand hygiene knowledge, attitudes, and practices among healthcare workers in Quetzaltenango, Guatemala in the COVID-19 context

**DOI:** 10.1371/journal.pgph.0004546

**Published:** 2025-05-14

**Authors:** Paulina Garzaro, Natalie Fahsen, Michelle M. Pieters, Christina Craig, Caroline Q. Pratt, Matthew J. Lozier, Celia Cordon-Rosales, Douglas R. Call, Brooke M. Ramay

**Affiliations:** 1 Center for Health Studies, Universidad del Valle de Guatemala, Guatemala City, Guatemala; 2 Division of Foodborne, Waterborne, and Environmental Diseases, National Center for Emerging and Zoonotic Infectious Diseases, Centers for Disease Control and Prevention (CDC), Atlanta, Georgia, United States of America; 3 Paul G. Allen School for Global Health, Washington State University (WSU), Pullman, United States of America; University of Michigan, UNITED STATES OF AMERICA

## Abstract

Healthcare workers and patients are at continual risk for healthcare-associated infections due to poor hand hygiene. Programs that support appropriate hand hygiene practices may mitigate this risk, although implementation is challenged by several barriers, including limited availability of hand hygiene products at the point of care, as described by the world health organization (WHO). In this study, we used mixed methodologies, including in-depth interviews and surveys to assess the knowledge, attitudes, practices, and barriers to appropriate hand hygiene practices among healthcare workers from 19 public primary and secondary healthcare facilities in Quetzaltenango, Guatemala. The qualitative data analyzed by thematic axes and quantitative data are described. We found that healthcare workers have a strong understanding of the importance of hand hygiene and how it contributes to patient health. We encountered knowledge gaps about the route of transmission of pathogens and how to prevent spread. Nevertheless, healthcare workers acknowledged the importance of training programs to strengthen hand hygiene practices. Potential barriers to improving hand hygiene practices included a lack of adequate infrastructure and resources for practicing hand hygiene with alcohol hand sanitizer, soap, and water. The results of this evaluation provide useful information for supporting hand hygiene practices in participant healthcare facilities and contributes to efforts to reduce the risks of healthcare-associated infections. Our findings likely apply to local healthcare facilities in other low- and middle-income countries and may be used to design hand hygiene educational materials for healthcare workers.

## Background

Healthcare workers (HCWs) and patients are at increased risk of infection with SARS-CoV-2 and other healthcare-associated infections due to regular patient contact [1]. Appropriate hand hygiene (HH) plays a critical role in the prevention of disease transmission and reduces healthcare-associated infections by up to 55% [2,3]. Proper HH, defined as handwashing with soap and water or sanitizing with alcohol-based hand rub (ABHR), should be carried out during five essential moments including before physical contact with patients, before an aseptic procedure, after actual or potential body fluid exposure, after physical contact with patients, and after contact with patient surrounding [3]. There are, however, several challenges to carrying out proper HH, including adherence to protocols, and sustainability of HH practices putting the safety of both patients and HCWs at risk [3].

Despite the perceived simplicity of HH practices and the availability of HH guidelines and protocols, adherence among HCWs remains low. Studies conducted in Syria and Egypt, which are low- and lower-middle-income countries, respectively, have reported poor HH practices during patient care [4,5]. The compliance with HH recommendations in HCFs has been shown to be sub optimal worldwide, with 64.5% in high-income countries and 9.1% in low-income countries [6].To address barriers to non-adherence, assessments of HH knowledge, attitudes, and practices (KAP) have been used to inform interventions to improve HH practices among HCWs [4,7–9]. Furthermore, periodic evaluation of HH in HCF provides a comprehensive approach to addressing HH barriers and can be relatively straightforward to assess using standardized guidelines provided by the World Health Organization (WHO) [10].

Studies conducted in Low- and Middle-income Countries (LMICs) have shown that poor HH adherence is often related to a lack of basic knowledge regarding cross-transmission of pathogens, incorrect use of ABHR, incorrect handwashing technique, and lack of attention to HH at five essential moments: before touching a patient, before a clean/aseptic procedure, after bodily fluid exposure risk, after touching a patient, and after touching patient surroundings [2,5,11]. Several studies emphasize the importance of systematic training and support from role models within the work area to promote a positive attitude toward and correct practice of HH. Unfortunately, barriers such as lack of infrastructure (e.g., hand-hygiene stations in the rooms where patients are treated) and the scarcity of resources, including water, soap, and ABHR, tend to impede adequate HH adherence in LMIC and are variables independent of human behavior [2,5]. And while ABHR is more effective against most microbes, soap and water should be used if *Clostridioides difficile*, Norovirus, or *Cryptosporidium* may be present or if hands are visibly dirty. Although seemingly simple, steps to truly improve HH can be complex, and sustainability can be elusive [8].

Where infrastructure is not a limiting factor, the sustainability of good HH practices depends on implementing culturally and contextually appropriate interventions that can be designed after identifying gaps [7,8]. In Guatemala, approximately 70% of the population receives free health services from public healthcare facilities (HCFs) [12], reinforcing the importance of understanding HH practices among HCWs from the public sector. There are currently no national infection prevention control (IPC) guidelines, and efforts to improve IPC are carried out by each individual clinic or hospital (personal communication). However, we previously evaluated HCWs’ HH practices in Quetzaltenango, Guatemala, before and after distributing ABHR to 19 public HCFs. Observations of 543 patient interactions demonstrated low adherence (40% before and 35% after ABRH distribution) with appropriate HH more frequently carried out during invasive procedures, after patient contact, and among physicians. The study highlighted contextual variations in adherence, underscoring the need to address multiple factors to improve HH practices and our knowledge, is the first publishing data from Guatemala [13]. Here, we carried out a cross-sectional, mixed methods study to identify the KAP of HH among HCWs during patient care to provide a broader understanding of HH practices in this setting.

## Methods

### Ethics statement

This study was reviewed and approved by the Research Ethics Committee of the Center for Health Studies of University del Valle, under Protocol No. 219-11-2020. The committee approved a waiver of signed consent and allowed investigators to carry out verbal informed consent for study participation with all study participants.

### Enrollment and data collection

During January 2021 to July 2023, we distributed locally produced ABHR to 19 HCFs located in the municipalities of San Juan Ostuncalco, San Miguel Sigüilá, Concepción Chiquirichapa, Nuevo Palmar, and Cantel, all located in the Quetzaltenango Department of Guatemala. Of the 19 HCFs, 14 were health posts (primary HCFs), four were health centers (secondary HCFs), and one was a permanent attention health center (secondary HCF). These facilities differ in the services they provide, the clinic hours, and the size of the population they serve. Secondary health centers provide the broadest range of medical services including lab testing, emergency assistance, general surgeries, general pediatrics, internal medicine, and other sub-specialties. Patients are usually referred to secondary healthcare centers by primary HCFs. Permanent attention health centers provide services 24-hours a day and include capacity to support maternal labor and delivery, and triage requiring overnight stays. Primary healthcare centers provide community-based preventive medicine services and promote public health. These facilities provide basic healthcare services such as vaccination, health check-ups, chronic disease treatment, treatment for acute minor illnesses, and basic pre-natal care.

A cross-sectional, mixed methods approach was used to characterize HH KAP among HCWs from these 19 HCFs during which time ABHR distribution was ongoing. Qualitative data were collected through in-depth, semi-structured interviews (for details see Questionnaire A, in S1 Text) with clinical staff from six facilities selected by convenience, while quantitative data were collected through a KAP survey (for details see Questionnaire B, in S1 Text) administered to staff at all facilities. In-depth interviews were carried out in June 2021, and participants were recruited and selected by convenience sampling. The interviews were conducted to understand HCWs’ perceptions of HH during patient care and were the starting point for the design of the KAP survey. Verbal consent was obtained for each interview participant, including permission to record the interview. Interviews were conducted in person, in Spanish, and were recorded, de-identified, transcribed, and translated into English. *MaxQDA* [14] software was used to code and analyze the interviews, in which seven key thematic areas were identified.

The KAP survey was administered to HCWs who 1. had direct contact with patients; 2. used a personal smartphone; and 3. had internet access. The survey was adapted from previously published KAP HH surveys [15–18]. Before survey administration, two members of the Quetzaltenango public health department reviewed and validated the questionnaire recommending clarification of some terms, such as the definition of “invasive” and “non-invasive procedures”. They also provided technical recommendations, including simplifying questions to improve comprehension. The KAP survey consisted of five sections: 1. demographic information; 2. evaluation of knowledge; 3. Attitudes; 4. practices regarding HH; and 5. barriers to HH compliance (see Tables A–E, in S1 Text). The survey did not specify in which situations ABHR or handwashing may be most effective, rather, questions were used to gauge general knowledge about effectiveness according to the type of hand hygiene evaluated. With the support of the directors of the participating HCFs, HCWs were provided a link via a messaging application to log into the anonymous KAP survey implemented through *KoBoToolbox* [19]. The survey was open for five months, from September 2021 to January 2022, during which time five reminders were sent both by the research team and by the district nurses and managers. If requested, surveys were conducted by telephone, which occurred in only four instances.

Descriptive statistics were generated using STATA [20], version 17.0. HCWs’ scores were tabulated for the knowledge section with a total of seven possible points, where one point was given for each correct answer. Knowledge questions were separated into two categories: 1. knowledge about HH practices; and 2. knowledge of the rationale underlying HH best practices. The knowledge score is presented and is the result of points earned from the two knowledge sections. A dichotomous variable was created to categorize nursing professions (nurse technicians and licensed nurses). A Mann-Whitney U test was used to assess differences between knowledge scores of HCWs at different healthcare levels (primary versus secondary healthcare levels) and between different types of nurses. Differences between physicians were not compared due to low response rate. Differences were considered statistically significant if *P* < 0.05.

## Results

A total of 10 in-depth, semi-structured interviews were conducted with healthcare personnel. Eighty HCWs were recruited to respond to the KAP survey, of which 77 met inclusion criteria and 38 completed the KAP survey (49%, n = 38/77) (Table 1).

**Table 1:** Characteristics of in-depth interview (IDI) and knowledge, attitudes, and practices (KAP) survey respondents.

Characteristics	IDI [N = 10] n (%)	KAP [N = 38] n (%)
Occupation	–	
*Physician*	0 (0)	3 (8)
*Laboratory Technician*	0 (0)	1 (3)
*Physician trainee*	2 (20)	0 (0)
*Nurse technician*	6 (60)	17 (45)
*Licensed Nurse*	2 (20)	17 (45)
Healthcare facility level where respondents work	–	
*Health Post*	4 (40)	22 (58)
*Health Center*	4 (40)	11 (29)
*Permanent Attention Health Center*	2 (2)	5 (13)

### Knowledge of HH practices

More than half of the KAP survey respondents (63%, n = 26/38) perceived application of ABHR as a faster method for HH than handwashing; 5% (n = 2/38) correctly identified ABHR as more effective against killing microorganisms than handwashing [3,21]. More than half of the KAP respondents (61%, n = 23/38) correctly indicated that the minimum handwashing duration with soap and water is 20 seconds [22]. Approximately half the respondents correctly indicated that HH at all five key moments is necessary to prevent the transmission of microorganisms to the patient (47%, n = 18/38) and to the health personnel (53%, n = 20/38) [22].

Interview respondents emphasized that the workplace plays an important role in HH education. Some mentioned the importance of receiving regular HH training from their supervisors to maintain good practice. The majority of KAP respondents (58%, n = 22/38) indicated that they had recently received HH training at the HCF.

### Knowledge of the rationale underlying HH best practices

About half the KAP survey respondents (55%, n = 21/38) correctly identified HCWs’ unwashed hands as the main route of transmission of microorganisms between patients and HCWs in health centers [3,8,21,23]. Only 26% (n = 10/38) correctly indicated the microorganisms causing healthcare-associated infections within the health center most frequently come from the patients themselves at the time of a healthcare visit which pose additional risk of contamination between patients and HCWs in the absence of proper HH procedures [3].

#### HH knowledge score.

**Fig 1:**
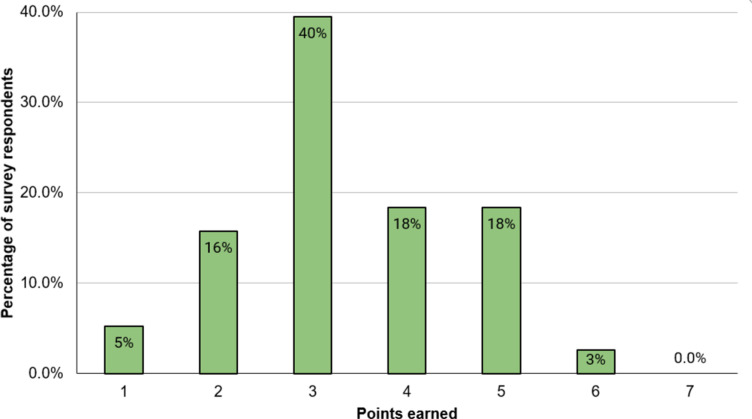
Distribution of hand hygiene knowledge scores of healthcare workers (n = 38) at 19 healthcare facilities in Quetzaltenango.

One respondent (3%; n = 1/38) earned more than 5 of 7 possible points from HH knowledge scale (Fig 1), while 37% (n = 14/38) earned 4 or 5 points, and the remaining 61% (n = 23/38) of respondents earned <4 points. The average knowledge score was 3 with a standard deviation of 1. There was no significant difference in knowledge scores when comparing healthcare level categories or licensed nurses with nurse technicians.

### Attitudes towards HH practices during patient care

#### Motivators of HH practice.

In the KAP survey, 47% (n = 18/38) of HCWs reported prevention of transmission of microorganisms from HCFs to community members as the most important reason for HH. Some (24%, n = 9/38) considered HH important to prevent transmission from the community to the HCFs. Additionally, 26% (n = 10/38) considered HH necessary to prevent healthcare-associated infections.

Interviews revealed that HH motivations were linked to a deep sense of ethical clinical practice and a commitment to ensure patient safety. HH raised HCWs’ confidence, as they felt that these practices protected them as well as their family members and other community members from becoming infected by microorganisms, viruses, and other microorganisms present in the HCFs. They also mentioned satisfaction from providing quality healthcare. The following quotes reflect their views on the subject:

***“I have a professional responsibility, and I do it [hand hygiene] to prevent getting infected.”***

(Auxiliary nurse 2, Health Center, 2021)

***“My coworkers, when they wash their hands using soap and water, know they are taking care of themselves and their patients.”***

(Professional nurse 2, Health Post, 2021)

#### Perceptions regarding the level of effort needed to practice proper HH.

Differences were reported in the perceived effort required to carry out proper HH practice during patient care. For example, 50% (n = 19/38) of KAP respondents indicated no real effort is needed, 40% (n = 15/38) felt a lot of effort is needed, and 11% (n = 4/38) felt it required some effort. Interviewees reported that lack of resources and high patient care demand made it harder to practice HH. The following quotes exemplify scenarios where HH would be omitted or replaced by alternative practices because of an emergency.

***“The only reason I would not wash is if we had no water because at times water runs out.”***

*(Auxiliary Nurse 6, Health Center, 2021)*

*“* ***When people come in critical there has been an accident, and we are rushing to do a procedure I just put on a new pair of gloves.”***

*(Auxiliary Nurse 4, Permanent Attention Health Center, 2021)*

### HH practices during patient care

#### Adherence.

According to the KAP survey respondents, handwashing with soap and water and the use of ABHR were common practices during patient care. A high percentage of respondents reported always performing HH after (74%, n = 28/38) and before (68%, n = 26/38) patient contact. Perceived high HH adherence was also emphasized in the following excerpt from interviews:

***“We also wash our hands after each procedure, after handling a child's [vaccine] card, after handing over medicine to a patient, and when patients hand us test results”.***

(Nurse 1, Health Post, 2021)

The COVID-19 pandemic was also cited by interviewees as impacting HCWs’ HH practices. For instance, interviewees perceived the need for HH when working with COVID-19 patients and during vaccination procedures.

#### Types of hand hygiene.

According to the KAP survey, HCWs practice different types of HH (ABHR and/or handwashing) depending on type of patient interaction. Though the number of respondents completing this set of questions varied, HCWs reported carrying out handwashing with soap and water for invasive procedures, such as after administering injections (47%, n = 17/36), after taking blood samples (53%, n = 19/38), or before treating wounds (60%, n = 21/35). ABHR use was reported during non-invasive procedures, such as before taking vital signs (80%, n = 28/35) and before measuring height and weight (69%, n = 25/36).

KAP respondents emphasized the importance of resource availability and time limitations as factors influencing the type of HH performed. Qualitative data demonstrated that participants reported using ABHR as an alternative when potable water is unavailable (due to decreased flow and/or water administration issues), when there are no handwashing stations in the patient room, or when there is not enough time to wash hands between patients. However, some HCWs reported discomfort from ABHR because of having sticky and/or dry hands after use. Furthermore, some respondents expressed uncertainty about the quality of ABHR.

***“I am not sure if the antibacterial gel [we are provided with] is good quality and should be used regularly as a substitute to handwashing. That is why I think washing my hands with water and soap is the best option, it makes me feel more comfortable.”***

(Physician 1, Health Post, 2021)

#### Barriers to HH practice.

KAP survey results demonstrate that the most common reasons for not practicing HH were linked to no handwashing stations in patient care rooms (40.5%, n = 15/37), no water available in handwashing stations (40.5%, n = 15/37), no soap available (16.2%, 6/37), or no ABHR available (16.2%, n = 6/37) (Table 2).

**Table 2:** Distribution of KAP survey responses for not practicing hand hygiene.

Data collected from healthcare workers (n = 37) from 19 healthcare facilities located in Quetzaltenango Department, Guatemala.	
Reasons for not practicing hand hygiene.	n (%) (N = 37)^*^
*Lack of handwashing stations in rooms for patient care*	15 (41)
*Lack of water*	15 (41)
*Lack of soap*	6 (16)
*Lack of ABHR*	6 (16)
*Lack of resources for drying hands*	6 (16)
*Lack of time/high workload*	4 (11)
*The products used for HH cause irritation and dryness*	2 (5)
*Not necessary when only touching the patient's skin*	1 (3)

* Multiple responses for N = 37 respondents, one person did not answer this question.

Interviewees mentioned that the limited budget for HH supplies and the need to install adequate water systems complicate their ability to practices appropriate HH. Scarcities of HH supplies were exacerbated during the pandemic and as a result, supplies were donated from non-governmental organizations, community leaders, local authorities, and the private sector. Additionally, interviewees reported occasionally purchasing HH supplies themselves. Even though HCWs assumed these responsibilities, they expressed the importance of strengthening HCF management for the procurement of donations and to work with personnel to carry out acquisition processes.

***“Not having supplies means an extra cost for us, because we must then buy the supplies ourselves to use them here at work.”***

(Auxiliary Nurse 6, Health Center, 2021)

***“Sometimes we talk to the Community Councils for Urban and Rural Development (COCODES) in Concepción Chiquirichapa, and ask them to bring us water.*”**

(Auxiliary Nurse 2, Health Center, 2021)

***“We have good auxiliary and professional nurses, and they are good at their jobs, but they are not managers. (...) so what do we do? We become managers.”***

(Nurse 1, Health Post, 2021)

## Discussion

We carried out a mixed methods study to assess HCWs’ KAP of HH during patient care. We identified barriers to practicing HH including lack of resources and HCW HH knowledge. Motivators contributing to HH practice were linked to perceived social norms associated with participants roles as healthcare providers.

According to HCWs, ethical and professional standards are embodied through HH best practices and associated with the ability to provide better healthcare. Comments made about the COVID-19 pandemic and perceived risk reflect the heightened awareness of the importance of consistently following the proper HH steps during disease outbreaks and when working with patients known to have highly contagious infections, like COVID-19. There appears to be genuine interest in and willingness to improve HH, and the importance of HH’s impact on human health is recognized by the participants. Similar studies have found that nurses perceptions of patient safety and the health benefits of practicing HH are the main motivators for reducing healthcare-associated infections, which can be linked to a commitment to providing a high standard of care [24]. Additionally, this study found that some motivations were linked to self-protection, potentially putting self-protection over that of the patient. This aligns with one study on the perspectives of HCWs from Belize during the COVID-19 pandemic, showing that adherence to HH was driven by self-care, serving as a means to protect both themselves and their families [25]. These positive attitudes support HCWs’ HH adherence [26].

In addition, HCWs in this study emphasized the role of HH in preventing transmission of microorganisms from the HCF to the community, and from the community to the HCF. The perceived risk of infection is an important driver of proper HH, has been reported in similar studies, and is characteristic of the experiences and backgrounds of healthcare providers [11,26].There is a strong perception that interaction with patients plays a role in the transmission of disease that can be prevented through correct HH at key moments.

Although preventing the transmission of microorganisms was a motivator for HH, less than half of HCW understood that HH is necessary at all five key moments to prevent microorganism transmission. Challenges in the ability to identify the main source of microorganisms within HCFs is a common problem among healthcare personnel [9,27,28]. Training programs that aim to strengthen and update knowledge by focusing on the importance of preventing disease transmission have been effective in the reduction of healthcare-associated infections within HCFs [9,11,26,27], and may also be effective in the HCFs included in this study.

HCWs expressed a preference for handwashing with soap and water over ABHR, reporting irritation and dryness after ABHR use. To overcome barriers to ABHR use, periodic training has been successful in improving adherence by demonstrating the positive impacts of ABHR on the prevention and reduction of healthcare-associated infections within HCFs, as well as proper use to avoid adverse effects [11]. Different formulations of ABHR may help reduce the physical discomfort, including the WHO-approved formulations, whose effectiveness and acceptability have been proven among HCWs. [21,29].

HCWs reported the effectiveness of ongoing training programs to improve knowledge around how and when to perform HH. Both work experience and HH training programs influence the knowledge and practices of HCWs [24,28]. Some studies show that access to continuing education on HH represents an opportunity for professional development at both the individual and facility levels, improving the overall quality of the healthcare provided [27], Therefore, consistent and sustainable training is a critical element that promotes HCWs to perform HH properly in the long term [27,28]. The incorporation of intervention models with multifaceted, evidence-based approaches to improve HH has been shown to be effective for improving HH practices among HCWs (25), (27). According to the WHO, these efforts should be integrated into multimodal interventions that address key factors influencing HCWs’ adherence and proper HH practices [10].

Potential barriers to improving HH were reported by participating HCWs, including a lack of adequate infrastructure and resources for practicing HH. The most common issues mentioned were the lack of water and hand-washing stations inside the rooms where patients are treated, and the inadequate supply of ABHR, both of which may be related to the Ministry of Health´s limited budget. Barriers related to HH infrastructure are frequently reported in LMICs and are attributed to the inability of the government to provide resources [9,11]. In our study, we found that HCWs actively compensate for the lack of consistent HH resources by collecting and managing donations from municipal and community organizations and private companies, and by purchasing HH materials with personal funds, but it is not clear if supply issues or other factors are driving behavior. Gaining clarity regarding the sustainability and effectiveness of these actions and how they can be supported is important to ensure adequate HH.

## Limitations

This study is subject to several limitations. First, the self-administered electronic surveys had low response rates. To overcome this limitation, nurses in charge at HCFs periodically reminded HCWs to respond to the KAP survey and were also available to resolve technical problems and respond to general questions about the surveys. Second, this was a local study and as such, generalizability to other HCFs, including others throughout Guatemala, cannot be assumed. Third, the assessment questions were specifically designed for this study and have not been previously validated in other studies, which may affect the generalizability and reliability of the findings. And finally, there is a risk of bias in the interviews and surveys conducted, including selection bias and social desirability bias. Exploring new tools and methodologies, including an in person survey, to gain clarity on KAP related to HH could help improve methods to continue to describe the local HH context.

## Conclusion

Improving access to HH materials and HH practices within the participating HCFs is an ongoing challenge that needs to be addressed. Gaps in basic knowledge, negative perceptions about ABHR use, lack of resources, and inadequate infrastructure were the key barriers highlighted by participants in our study. Our findings can be used as a starting point for designing strategies to ensure the acceptability and effectiveness of possible solutions to improve HH practices. Perceptions presented here, such as motivations, represent potential behavioral drivers that can be emphasized during implementation of strategies designed to improve HH [2,9,27]. Based on the results of this study, the authors are already working with the Guatemalan Ministry of Health to address HH barriers including supply of ABHR, and the design of educational materials to support HH training, strengthen HH knowledge, and promote HH adherence at key moments.

More research in the field is needed to explore additional factors that may influence the improvement of HH after these interventions are implemented and evaluated. In addition, triangulating these KAP findings with ongoing situational analyses using for example the WHO Hand Hygiene Self-Assessment Framework (HHSAF) [30] would support ongoing development of HH practices.

## Supporting information

S1 Text In-depth interview questions, and survey responses for questionnaire to assess the knowledge, attitudes, practices, and barriers to appropriate hand hygiene practices among healthcare workers Guatemala. (DOCX)

S1 Data Original questionnaire dataset. (XLS)

S1 Checklist Inclusivity in global research. (DOCX)
