# Supplementary material for: Qualitative and quantitative evaluation of hand hygiene knowledge, attitudes, and practices among healthcare workers in Quetzaltenango, Guatemala in the COVID-19 context
Source: PLOS Glob Public Health. 2025 May 14;5(5):e0004546. doi: 10.1371/journal.pgph.0004546 (PMC12077707; doi:10.1371/journal.pgph.0004546)
Supplement: S1 Text — (DOCX) [file pgph.0004546.s001.docx]

**Qualitative and quantitative evaluation of hand hygiene knowledge, attitudes, and practices among healthcare workers Quetzaltenango, Guatemala in the context of COVID-19**

Paulina Garzaro¹, Natalie Fahsen¹, Michelle M. Pieters¹, Christina Craig², Caroline Q. Pratt², Matthew J. Lozier², Celia Cordon-Rosales¹^,3^, Douglas R. Call³, and Brooke M. Ramay^1,3,*^

¹Center for Health Studies, Universidad del Valle de Guatemala, Guatemala City, Guatemala

²Division of Foodborne, Waterborne, and Environmental Diseases, National Center for Emerging and Zoonotic Infectious Diseases, Centers for Disease Control and Prevention (CDC), Atlanta, USA

³Paul G. Allen School of Global Health, Washington State University (WSU), Pullman, USA

Supplementary material

This document presents the in depth interview questions. The second section includes the complete set of questions and corresponding answers from the KAP hand hygiene survey, completed by 38 healthcare workers across 19 healthcare facilities in the department of Quetzaltenango, Guatemala.

**Questionnaire A. Semi-Structured Interview: Healthcare Workers' Knowledge, Perceptions, and Practices on Hand Hygiene.**

Interview Guide

**Section I: General Information**

1. Healthcare facility where you work:
2. Municipality and village where the facility is located:
3. Your profession:
4. Your role within the healthcare facility:
5. How long have you been working at this facility?

**Section II: Knowledge, Attitudes, and Practices Related to Hand Hygiene**

Hand Hygiene Practices:

- What HH methods are you familiar with in your practice?
- During a typical workday, which HH methods do you use? Why?
- What are your preferred HH products? Why?
- What motivates you to practice HH during a regular workday?
- In what situations do you practice HH during patient care?
- How do HH practices change during invasive vs. non-invasive procedures?
- What do you do when you touch only the patient's surroundings (e.g., door handles, bedding) without direct patient contact?

Perceived Benefits of Hand Hygiene:

- Do you believe HH has benefits? What are they?
- What are the benefits of HH for you? For patients and other staff?
- Do these benefits differ between invasive and non-invasive procedures? How?

Emotional and Physical Response to Hand Hygiene:

- How do you feel physically and mentally when practicing HH? Why?

Cues for Hand Hygiene Action:

- What reminders prompt you to practice HH during a regular workday?
- Are there visual cues that encourage HH?
- Do these cues change for invasive vs. non-invasive procedures?

Facilitators and Barriers to Hand Hygiene:

- What factors within your healthcare facility make it easier for you to practice HH?
- What challenges or barriers prevent you from practicing HH?
- What situations make HH difficult in your daily work?
- What could help overcome these barriers?
- Are there specific barriers for invasive vs. non-invasive procedures?

Situations Where Hand Hygiene is Omitted:

- Can you provide an example of a situation where you should have performed HH but omitted it? Why?
- What factors contributed to skipping HH in that situation?

Perceived Benefits of Not Practicing Hand Hygiene:

- Do you think there are benefits to not practicing HH in certain situations? What are they?
- What benefits, if any, do you experience when not performing HH?
- How does this impact others?

Alternative Behaviors to Hand Hygiene:

- In situations where you do not perform HH, what do you do instead? Why?

**Section III: Use of Alcohol-Based Hand Rub (ABHR)**

- Do you use ABHR at work? When and where?
- Why do you choose ABHR over other HH methods?
- What do you do when an ABHR dispenser is broken or empty?
- Who is responsible for refilling or repairing ABHR dispensers?
- Do you use handwashing stations at work? When and where?
- Why do you use handwashing stations instead of other HH methods?
- Have you ever requested more ABHR when it was running low?
- Have you ever encountered issues accessing ABHS at your healthcare facility?

**Section IV: Perceptions of Hand Hygiene Practices in the Facility**

- What does good HH practice mean to you?
- Where did you learn about proper HH practices?
- How would you describe the HH practices of staff at your facility?
- How can healthcare workers contribute to improving HH practices?
- What actions would help enhance HH practices at your facility?

**Questionnaire B. KAP hand hygiene survey and distribution of responses**

**Table A.** **Distribution of responses to survey questions - General information**

| **Question** | **n (%)** |
| --- | --- |
| **Date** |  |
| 10th of September 2021 - 18th of January 2022 | |
| **Gender (N = 38)** |  |
| *Femenine* | 31 (81.6) |
| *Masculine* | 7 (18.4) |
| **How long have you been working in this healthcare facility? (N = 35)** |  |
| *Less than a year* | 3 (8.6) |
| *Between 1 and 5 years* | 14 (40.0) |
| *Between 6 and 10 years* | 10 (28.6) |
| *More than 10 years* | 8 (22.9) |
| **Occupation (N = 38)** |  |
| *Physician* | 3 (7.9) |
| *Licensed Nurse* | 17 (44.7) |
| *Nurse technician* | 17 (44.7) |
| *Laboratory Technician* | 1 (2.6) |
| *Nurse trainee* | 0 (0.0) |
| *Physician trainee* | 0 (0.0) |
| *Other* | 0 (0.0) |
| **Healthcare facility level where you work (N = 38)** |  |
| *Permanent Attention Health Center* | 5 (13.2) |
| *Health Center* | 11 (28.9) |
| *Health Post* | 22 (57.9) |
| **Municipaliy (N = 38)** |  |
| *Cantel* | 14 (36.8) |
| *Concepción Chiquirrichapa* | 5 (13.2) |
| *Nuevo Palmar* | 11 (28.9) |
| *San Juan Ostuncalco* | 3 (7.9) |
| *San Miguel Sigüilá* | 5 (13.2) |
| **Village (N = 37)** |  |
| *Agua Blanca* | 1 (2.7) |
| *Aldea Monrovia* | 1 (2.7) |
| *Cantel* | 6 (16.2) |
| *Chirijquiac* | 2 (5.4) |
| *Chuisuc* | 1 (2.7) |
| *Concepcion Chiquirichapa* | 2 (5.4) |
| *Duraznales* | 1 (2.7) |
| *La Emboscada* | 3 (8.1) |
| *La Estancia* | 1 (2.7) |
| *Nuevo Palmar* | 5 (13.5) |
| *San Juan Ostuncalco* | 1 (2.7) |
| *San Marcos* | 3 (8.1) |
| *San Miguel Siguila* | 2 (5.4) |
| *Telena* | 1 (2.7) |
| *Tuipox* | 1 (2.7) |
| *Xecam* | 1 (2.7) |
| *Pachaj* | 1 (2.7) |
| *Pasac* | 1 (2.7) |
| *Calahuache* | 3 (8.1) |

**Table B. Distribution of responses to survey questions - Knowledge**

| **Question** | **n (%)** |
| --- | --- |
| **Have you received hand hygiene training? (N = 38)** |  |
| *No* | 1 (2.6) |
| *Yes* | 37 (97.4) |
| **When was the last hand hygiene training you received? (N = 38)** |  |
| *Less than a year ago* | 22 (57.9) |
| *A year ago* | 6 (15.8) |
| *More than a year ago* | 9 (23.7) |
| *Other* | 1 (2.6) |
| *Don't know/No opinion* | 0 (0.0) |
| **If other, please specify (N = 1)** |  |
| *Never* | 1 (100.0) |
| **Which of the following is the main route of cross-transmission of potentially pathogenic microorganisms between patients in healthcare facilities? (N = 38)** |  |
| *Healthcare professionals' hands when they are not clean* | 21 (55.3) |
| *The air circulating in the health center* | 1 (2.6) |
| *Exposure of patients to germ-colonized surfaces* | 12 (31.6) |
| *Sharing non-invasive objects between one patient and another* | 2 (5.3) |
| *Other* | 2 (5.3) |
| *Don't know/No opinion* | 0 (0.0) |
| **If other, please specify (N = 2)** |  |
| *All of the above* | 2 (100.0) |
| **What is the most frequent source of germs causing healthcare-associated infections within the health center where you work? (N = 38)** |  |
| *The water system* | 13 (34.2) |
| *The air* | 0 (0.0) |
| *Microorganisms already present in the patient* | 10 (26.3) |
| *The surfaces* | 14 (36.8) |
| *Other* | 1 (2.6) |
| *Don't know/No opinion* | 0 (0.0) |
| **If other, please specify (N = 0)** |  |
| *Lack of water and handwashing stations* | 1 (100.0) |
| ***Which of the following hand hygiene actions prevents the transmission of microorganisms to the patient? (N = 38)** |  |
| *Before patient contact* | 37 (97.4) |
| *Immediately after the risk of exposure to bodily fluids* | 27 (71.1) |
| *After contact with the patient's close environment* | 22 (57.9) |
| *After patient contact* | 30 (78.9) |
| *Immediately before an aseptic procedure* | 25 (65.8) |
| *Other* | 0 (0.0) |
| *Don't know/No opinion* | 0 (0.0) |
| **If other, please specify (N = 0)** |  |
| ***Which of the following hand hygiene actions prevents the transmission of microorganisms to the healthcare personnel? (N = 38)** |  |
| *Before patient contact* | 29 (76.3) |
| *Immediately after the risk of exposure to bodily fluids* | 30 (78.9) |
| *After contact with the patient's close environment* | 27 (71.1) |
| *After patient contact* | 33 (86.8) |
| *Immediately before an aseptic procedure* | 26 (68.4) |
| *Other* | 0 (0.0) |
| *Don't know/No opinion* | 0 (0.0) |
| **If other, please specify (N = 0)** |  |
| ***Which of the following statements about ABHR use and handwashing with soap and water are true? (N = 38)** |  |
| *Use of ABHR is faster than handwashing* | 24 (63.2) |
| *Use of ABHR causes more skin dryness than handwashing* | 13 (34.2) |
| *Use of ABHR is more effective against germs than handwashing* | 2 (5.3) |
| *It is recommended that handwashing and use of ABHR be performed sequentially* | 24 (63.2) |
| *Handwashing is more effective against germs than ABHR use* | 18 (47.4) |
| *Other* | 0 (0.0) |
| *Don't know/No opinion* | 0 (0.0) |
| **If other, please specify (N = 0)** |  |
| **What is the minimum time required for ABHR to kill germs on hands?**  **(N = 38)** |  |
| *Less than 20 seconds* | 9 (23.7) |
| *20 seconds* | 15 (39.5) |
| *More than 20 seconds* | 14 (36.8) |
| *Other* | 0 (0.0) |
| *Don't know/No opinion* | 0 (0.0) |
| **If other, please specify (N = 0)** |  |
| **What is the minimum time required for handwashing with water and soap to kill germs on hands? (N = 38)** |  |
| *Less than 20 seconds* | 2 (5.3) |
| *20 seconds* | 13 (34.2) |
| *More than 20 seconds* | 23 (60.5) |
| *Other* | 0 (0.0) |
| *Don't know/No opinion* | 0 (0.0) |
| **If other, please specify (N = 0)** |  |
| **Should ABHR use practices be different if the clinical procedure is invasive or non-invasive? (N = 38)** |  |
| *No* | 16 (42.1) |
| *Yes* | 21 (55.3) |
| *Don't know/No opinion* | 1 (2.6) |
| **Should hand hygiene practices with soap and water be different if the clinical procedure is invasive or non-invasive? (N = 38)** |  |
| *No* | 14 (36.8) |
| *Yes* | 22 (57.9) |
| *Don't know/No opinion* | 2 (5.3) |
| **What impact does hand hygiene have on the prevention of healthcare-associated infections in patients? (N = 38)** |  |
| *Very low* | 1 (2.6) |
| *Low* | 3 (7.9) |
| *High* | 17 (44.7) |
| *Very high* | 17 (44.7) |
| ***Multiple responses allowed. Denominator is the total number of people that responded to the question** | |

**Table C.** **Distribution of responses to survey questions - Attitudes**

| **Distribution of responses to survey questions - Attitudes** | |
| --- | --- |
| **Question** | **n (%)** |
| **Do you think hand hygiene is important? (N = 37)** |  |
| *No* | 0 (0.0) |
| *Yes* | 37(100.0) |
| **Do you think hand hygiene practices with ABHR should be different depending on the type of clinical procedure: invasive and non-invasive? (N = 37)** |  |
| *No* | 17 (45.9) |
| *Yes* | 20 (54.1) |
| **Do you think hand hygiene practices with water and soap should be different depending on the type of clinical procedure: invasive and non-invasive? (N = 38)** |  |
| *No* | 15 (39.5) |
| *Yes* | 23 (60.5) |
| **What is the main reason for frequent hand hygiene at the healthcare facility? (N = 38)** |  |
| *Prevent infections acquired by patients at the healthcare facility* | 10 (26.3) |
| *Preventing the bacteria transfer from the healthcare facility to the community* | 18 (47.4) |
| *Preventing the bacteria transfer from the community to the healthcare facility* | 9 (23.7) |
| *Eliminate visible dirt* | 1 (2.6) |
| **During patient care, what priority is given to hand hygiene? (N = 36)** |  |
| *Low* | 1 (2.8) |
| *Moderate* | 2 (5.6) |
| *High* | 18 (50.0) |
| *Very high* | 15 (41.7) |
| **Are you constantly looking for ways to improve your hand hygiene practices? (N = 38)** |  |
| *No* | 4 (10.5) |
| *Yes* | 34 (89.5) |
| ***In what ways are you looking to improve your hand hygiene practices? (N = 28)** |  |
| *Practicing handwashing with soap and water correctly and consistently* | 14 (50.0) |
| *Constantly using ABRH* | 8 (28.6) |
| *Ensuring constant availability of the necessary supplies (e.g. ABHR, water, soap).* | 4 (14.3) |
| *Learning about correct use of ABHR and proper handwashing* | 4 (14.3) |
| *Other* | 2 (7.1) |
| **How important is it to your supervisor that you perform proper hand hygiene? (N = 36)** |  |
| *1. Unimportant* | 3 (8.3) |
| *2. Somewhat unimportant* | 2 (5.6) |
| *3. Neutral* | 3 (8.3) |
| *4. Important* | 12 (33.3) |
| *5. Very important* | 16 (44.4) |
| **How important is it to your coworkers that you perform proper hand hygiene? (N = 37)** |  |
| *1. Unimportant* | 1 (2.7) |
| *2. Somewhat unimportant* | 3 (8.1) |
| *3. Neutral* | 6 (16.2) |
| *4. Important* | 12 (32.4) |
| *5. Very important* | 15 (40.5) |
| **How important is it to the patients that you perform proper hand hygiene? (N = 37)** |  |
| *1. Unimportant* | 5 (13.5) |
| *2. Somewhat unimportant* | 4 (10.8) |
| *3. Neutral* | 5 (13.5) |
| *4. Important* | 11 (29.7) |
| *5. Very important* | 12 (32.4) |
| **How much effort do you need to perform good hand hygiene when providing care to patients? (N = 38)** |  |
| *1. No effort* | 14 (36.8) |
| *2. Almost no effort* | 5 (13.2) |
| *3. Neutral* | 4 (10.5) |
| *4. Effort* | 4 (10.5) |
| *5. A lot of effort* | 11(28.9) |
| ***Open-ended question. Denominator is the total number of people that responded to the question** | |

**Table D.** **Distribution of responses to survey questions - Practices**

| **Question** | **n (%)** |
| --- | --- |
| **Do you use ABHR for hand hygiene on a regular basis at healthcare facility where you work? (N = 36)** |  |
| *No* | 0 (0.0) |
| *Yes* | 36(100.0) |
| **Do you perform handwashing with soap and water on a regular basis at healthcare facility where you work? (N = 38)** |  |
| *No* | 3 (7.9) |
| *Yes* | 35 (92.1) |
| **What type of hand hygiene do you practice in the following situations?** |  |
| **Prior to taking vital signs (N = 36)** |  |
| *ABHR* | 25 (69.4) |
| *Handwashing with soap and water* | 4 (11.1) |
| *Both* | 7 (19.4) |
| *None* | 0 (0.0) |
| **Before measuring weight and height (N = 35)** |  |
| *ABHR* | 28 (80.0) |
| *Handwashing with soap and water* | 2 (5.7) |
| *Both* | 5 (14.3) |
| *None* | 0 (0.0) |
| **Before administering an injection (N = 36)** |  |
| *ABHR* | 13 (36.1) |
| *Handwashing with soap and water* | 12 (33.3) |
| *Both* | 10 (27.8) |
| *None* | 1 (2.8) |
| **Before taking a blood sample (N = 37)** |  |
| *ABHR* | 9 (24.3) |
| *Handwashing with soap and water* | 16 (43.2) |
| *Both* | 11 (29.7) |
| *None* | 1 (2.7) |
| **Before healing a wound (N = 35)** |  |
| *ABHR* | 4 (11.4) |
| *Handwashing with soap and water* | 21(60.0) |
| *Both* | 9 (25.7) |
| *None* | 1 (2.9) |
| **Before putting on gloves (N = 34)** |  |
| *ABHR* | 19 (55.9) |
| *Handwashing with soap and water* | 9 (26.5) |
| *Both* | 4 (11.8) |
| *None* | 2 (5.9) |
| **After putting on gloves (N = 35)** |  |
| *ABHR* | 14 (40.0) |
| *Handwashing with soap and water* | 12 (34.3) |
| *Both* | 7 (20.0) |
| *None* | 2 (5.7) |
| **After administering an injection (N = 36)** |  |
| *ABHR* | 10 (27.8) |
| *Handwashing with soap and water* | 17 (47.2) |
| *Both* | 8 (22.2) |
| *None* | 1 (2.8) |
| **After taking a blood sample (N = 36)** |  |
| *ABHR* | 4 (11.1) |
| *Handwashing with soap and water* | 19 (52.8) |
| *Both* | 12 (33.3) |
| *None* | 1 (2.8) |
| **After healing a wound (N = 37)** |  |
| *ABHR* | 5 (13.5) |
| *Handwashing with soap and water* | 20 (54.1) |
| *Both* | 11 (29.7) |
| *None* | 1 (2.7) |
| **After taking vital signs (N = 36)** |  |
| *ABHR* | 20 (55.6) |
| *Handwashing with soap and water* | 8 (22.2) |
| *Both* | 8 (22.2) |
| *None* | 0 (0.0) |
| **After measuring weight and height (N = 35)** |  |
| *ABHR* | 21 (60.0) |
| *Handwashing with soap and water* | 7 (20.0) |
| *Both* | 7 (20.0) |
| *None* | 0 (0.0) |
| **When entering and leaving a room (N = 36)** |  |
| *ABHR* | 25 (69.4) |
| *Handwashing with soap and water* | 7 (19.4) |
| *Both* | 4 (11.1) |
| *None* | 0 (0.0) |
| **How often do you perform hand hygiene (with ABHR or soap and water) BEFORE patient care? (N = 38)** |  |
| Never | 0 (0.0) |
| Sometimes | 0 (0.0) |
| Often | 12 (31.6) |
| Always | 26 (68.4) |
| **How often do you perform hand hygiene (with ABHR or soap and water) AFTER patient care? (N = 38)** |  |
| Never | 0 (0.0) |
| Sometimes | 1 (2.6) |
| Often | 9 (23.7) |
| Always | 28 (73.7) |
| **How often do your coworkers perform hand hygiene (with ABHR or soap and water) BEFORE patient care? (N = 37)** |  |
| Never | 0 (0.0) |
| Sometimes | 1 (2.7) |
| Often | 17 (45.9) |
| Always | 19 (51.4) |
| **How often do your coworkers perform hand hygiene (with ABHR or soap and water) AFTER patient care? (N = 37)** |  |
| Never | 0 (0.0) |
| Sometimes | 1 (2.7) |
| Often | 13 (35.1) |
| Always | 23 (62.2) |
| **Do you change the way you practice hand hygiene with ABHR depending on the type of clinical procedure you are going to perform: invasive and non-invasive? (N = 38)** |  |
| No | 26 (68.4) |
| Yes | 12 (31.6) |
| ****If yes, what practices change? (N = 7)** |  |
| *Handwashing with soap and water* | 4 (57.1) |
| *More thorough handwashing (e.g. with a surgical brush)* | 1 (14.3) |
| *Other* | 2 (28.6) |
| **Do you change the way you practice hand hygiene with water and soap depending on the type of clinical procedure you are going to perform: invasive and non-invasive? (N = 38)** |  |
| No | 24 (63.2) |
| Yes | 14 (36.8) |
| ****If yes, what practices change? (N = 10)** |  |
| *Handwashing with soap and water* | 1 (10.0) |
| *More thorough handwashing (e.g. with a surgical brush, extending the handwashing time)* | 4 (40.0) |
| *Other* | 5 (50.0) |
| ***If you sometimes do not practice hand hygiene before and after contact with a patient, what are the reasons for this? (N = 37)** |  |
| *The products used for hand hygiene cause irritation and dryness* | 2 (5.4) |
| *Handwashing stations are not available in all rooms where patients are treated* | 15 (40.5) |
| *Lack of water* | 15 (40.5) |
| *Lack of soap* | 6 (16.2) |
| *Lack of ABHR* | 6 (16.2) |
| *Lack of resources for drying hands* | 6 (16.2) |
| *Lack of time/high workload* | 4 (10.8) |
| *ABHR is not very effective* | 0 (0.0) |
| *Not necessary when hands are visibly clean* | 0 (0.0) |
| *Not necessary when only touching the patient's skin (superficially)* | 1 (2.7) |
| *I always practice hand hygiene before and after contact with patients* | 23 (62.2) |
| *Other* | 0 (0.0) |
| **If other, please specify (N = 0)** |  |
| ***Multiple responses allowed. Denominator is the total number of people that responded to the question **Open-ended question. Denominator is the total number of people that responded to the question** | |

**Table E. Distribution of responses to survey questions - Actions to improve hand hygiene**

| Question | n (%) |
| --- | --- |
| **How would you rate your hand hygiene practices during patient care? (N = 38)** |  |
| 1. Very bad | 1 (2.6) |
| 2. Bad | 0 (0.0) |
| 3. Neutral | 2 (5.3) |
| 4. Good | 18 (47.4) |
| 5. Very good | 17 (44.7) |
| **How effective do you think the following actions are for the permanent improvement of hand hygiene in the healthcare facility where you work?** |  |
| **For the directors of each Health District to constantly support and promote hand hygiene? (N = 38)** |  |
| 1. Ineffective | 2 (5.3) |
| 2. Somewhat ineffective | 0 (0.0) |
| 3. Neutral | 7 (18.4) |
| 4. Effective | 10 (26.3) |
| 5. Very effective | 19 (50.0) |
| **To have hand hygiene resources (ABHR or handwashing stations) in each room where patients are cared for? (N = 38)** |  |
| 1. Ineffective | 0 (0.0) |
| 2. Somewhat ineffective | 1 (2.6) |
| 3. Neutral | 5 (13.2) |
| 4. Effective | 8 (21.1) |
| 5. Very effective | 24 (63.2) |
| **To have hand hygiene posters at the point of care as a reminder? (N = 38)** |  |
| 1. Ineffective | 1 (2.6) |
| 2. Somewhat ineffective | 2 (5.3) |
| 3. Neutral | 6 (15.8) |
| 4. Effective | 7(18.4) |
| 5. Very effective | 22 (57.9) |
| **For all healthcare personnel to receive hand hygiene training? (N = 38)** |  |
| 1. Ineffective | 0 (0.0) |
| 2. Somewhat ineffective | 1 (2.6) |
| 3. Neutral | 5 (13.2) |
| 4. Effective | 7 (18.4) |
| 5. Very effective | 25 65.8) |
| **To have clear and simple materials on hand hygiene accessible to all health personnel? (N = 38)** |  |
| 1. Ineffective | 1 (2.6) |
| 2. Somewhat ineffective | 1 (2.6) |
| 3. Neutral | 7 (18.4) |
| 4. Effective | 9 (23.7) |
| 5. Very effective | 20 52.6) |
| **For all healthcare personnel to receive regular feedback on their hand hygiene performance? (N = 38)** |  |
| 1. Ineffective | 0 (0.0) |
| 2. Somewhat ineffective | 2 (5.3) |
| 3. Neutral | 5 (13.2) |
| 4. Effective | 13 (34.2) |
| 5. Very effective | 18 (47.4) |
| **Would you feel comfortable receiving feedback (on your hand hygiene practices) from your colleagues? (N = 38)** |  |
| No | 1 (2.6) |
| Yes | 37 97.4) |
| If the above answer is "no", please specify why (N = 1) |  |
| "This is a practice that all health personnel should be aware of and perhaps it should be observed how the staff does it, instead of training them" | 1 (100.0) |
| **Would you feel comfortable giving feedback (on your hand hygiene practices) to your colleagues? (N = 38)** |  |
| No | 1 (2.6) |
| Yes | 37 (97.4) |
| If the above answer is "no", please specify why (N = 1) |  |
| "The staff is in charge of providing handwashing training to users, thus the staff is knowledgeable on the subject" | 1(100.0) |
| **For you to perform hand hygiene perfectly (setting a good example for your colleagues)? (N = 38)** |  |
| 1. Ineffective | 0 (0.0) |
| 2. Somewhat ineffective | 0 (0.0) |
| 3. Neutral | 3 (7.9) |
| 4. Effective | 16 (42.1) |
| 5. Very effective | 19 (50.0) |
| **For patients to be invited to remind healthcare personnel to perform good hand hygiene? (N = 38)** |  |
| 1. Ineffective | 5 (13.2) |
| 2. Somewhat ineffective | 0 (0.0) |
| 3. Neutral | 8 (21.1) |
| 4. Effective | 9 (23.7) |
| 5. Very effective | 16 (42.1) |
| ***What other action can contribute to the improvement of hand hygiene practices of both you and your colleagues? (N = 28)** |  |
| Training (with demonstration) about:  -The positive impact of handwashing - Proper use of ABHR - Proper handwashing techniques | 9 (32.1) |
| Water availability | 7 (25.0) |
| Hand hygiene promotion | 5 (17.9) |
| ABHR availability | 3 (10.7) |
| Receiving feedback on handwashing | 3 (10.7) |
| Soap availability | 2 (7.1) |
| Receiving supervision | 1 (3.6) |
| Other | 6 (21.4) |
| **Open-ended question. Denominator is the total number of people that responded to the question | |
